# Modulation of tumor microenvironment for immunotherapy: focus on nanomaterial-based strategies

**DOI:** 10.7150/thno.42998

**Published:** 2020-02-10

**Authors:** Yun Liu, Jianfeng Guo, Leaf Huang

**Affiliations:** 1 Division of Pharmacoengineering and Molecular Pharmaceutics, Eshelman School of Pharmacy, University of North Carolina at Chapel Hill, Chapel Hill, North Carolina 27599, United States; 2 School of Pharmaceutical Sciences, Jilin University, Changchun 130021, China

**Keywords:** tumor immunology, characterization and quantification of immunoregulatory cells, nanoparticles, drug delivery, combination therapy

## Abstract

Recent advances in the field of immunotherapy have profoundly opened up the potential for improved cancer therapy and reduced side effects. However, the tumor microenvironment (TME) is highly immunosuppressive, therefore, clinical outcomes of currently available cancer immunotherapy are still poor. Recently, nanomaterial-based strategies have been developed to modulate the TME for robust immunotherapeutic responses. In this review, the immunoregulatory cell types (cells relating to the regulation of immune responses) inside the TME in terms of stimulatory and suppressive roles are described, and the technologies used to identify and quantify these cells are provided. In addition, recent examples of nanomaterial-based cancer immunotherapy are discussed, with particular emphasis on those designed to overcome barriers caused by the complexity and diversity of TME.

## 1. Introduction

Recent knowledge of the crosstalk between cancer cells and the host immune system (termed the cancer-immunity cycle) has opened up the potential for cancer immunotherapy 1. The clinical promise of different strategies such as monoclonal antibodies (mAbs, conjugated with and without drugs) 2, cancer vaccines 3, adoptive T cell therapy 4 and immune checkpoint inhibitors (mostly antibodies) 5, has underscored the status of immunotherapy as a pillar of cancer treatment. However, the neoplastic foci (unlike hematologic malignancies) is normally surrounded by immune cells, fibroblasts, soluble signaling molecules, blood vessels, and the extracellular matrix (ECM) (see more details in 6). These cells/components in the tumor microenvironment (TME) cause serious resistance to currently available immune-based therapies 6. For example, the adoptive transfer of genetically engineered T cells expressed with chimeric antigen receptors (CARs) has achieved promising results in the treatment of acute lymphocytic leukemia (one of blood cancers) with up to 90% of five-year overall survival, but this treatment has been significantly limited in solid tumors 7. Therapeutic efficacy of antibody-drug conjugates and cancer vaccines is also largely attenuated by immunosuppression caused by the TME 8. In addition, the blockade of immune checkpoint molecules (e.g. programmed cell death protein 1, PD-1; cytotoxic T lymphocyte-associated protein 4, CTLA-4) using antibodies has demonstrated great promise for sculpting tumor immunogenicity in certain solid tumors (e.g. melanoma and non-small cell lung cancer) 8; however, response rates to immune checkpoint inhibitors tremendously vary in different tumor types, which is mainly attributed to the complex nature of TME 9.

Recently, increasing research in nanomaterials has offered great potential for the improvement of cancer immunotherapy 10, but the immunosuppressive TME still limits the efficacy. Therefore, it is really of critical importance to understand the complexity and diversity of TME. Recent advances in technologies such as high-solution imaging, flow cytometry and next-generation sequencing are anticipated to provide a comprehensive view of TME constituents 6, which will inspire the development of novel nanoformulations to advance cancer immunotherapy. In this review, the immunoregulatory cells (cells relating to the regulation of immune responses) in terms of stimulatory and suppressive roles in the TME are described, and the techniques used to characterize and quantify them are provided. This review will also discuss different nanoparticle (NP) strategies under investigation for cancer immunotherapy, with specific emphasis on those designed for circumventing barrages caused by the TME.

## 2. Immunoregulatory Cell Types in TME

Tumorigenesis as a complex and dynamic process is generally comprised of three phases namely initiation, development and metastasis. The interactions between malignant/non-malignant cells and cellular/non-cellular components form a microenvironment surrounding the neoplastic foci 6. Inside there, the ECM (a complex network of proteins, proteoglycans and enzymes) provides the physical and biochemical support for surrounding cells, and the crosstalk between tumor cells, immune cells and stromal cells via the secretion of cytokines and chemokines causes the escape of immunosurveillance for tumor progression 11. The details of tumor/non-tumor cell communications and cell-ECM interactions have been substantially studied 12, which assist in understanding the structural and physiological obstacles associated with the TME and consequently improving cancer therapies. Generally, cells inside the TME 13 include: immune cells (e.g. dendritic cells, lymphocytes, macrophages and myeloid-derived suppressor cells (MDSCs)), cells of mesenchymal origin (e.g. fibroblasts, myofibroblasts, mesenchymal stromal cells), and vascular cells (e.g. endothelial cells and pericytes).

In this section, we will discuss the cell types within the TME in terms of immunostimulatory and immunosuppressive roles (Table 1) and describe technologies for characterization and quantification of these cells, hoping to understand the mechanisms of immunotherapy resistance, identify potential therapeutic targets, and advance antitumor immunity for long-term effects and/or eradication of cancer.

### 2.1. Immunostimulatory cells

**Figure 1:**
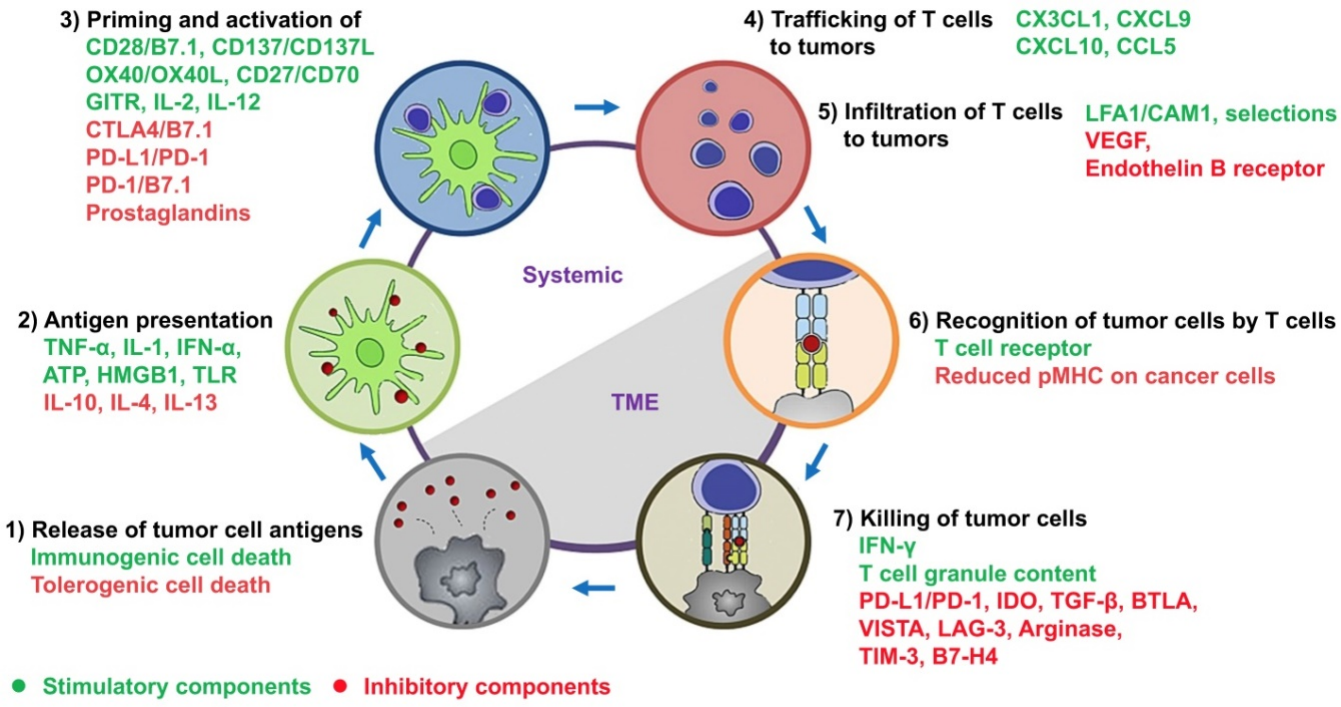
The cancer-immunity cycle in tandem with a summary of stimulatory and inhibitory components. As depicted by Chen and Mellman, this cycle is comprised of 1) release of tumor cell antigens by dying cancer cells, 2) antigen presentation by DCs, 3) priming and activation of T cells, 4) trafficking and 5) infiltration of activated T cells to tumors, 6) recognition of tumor cells by activated T cells, and 7) killing of tumor cells. The stimulatory and inhibitory factors together form an immune regulatory network for the modulation of cancer-immunity cycle. This figure has been modified from 1 and 10.

*Dendritic cells (DCs)*: It is known that a large number of genetic mutations and the failure of normal cellular regulatory processes are evident in cancers. These abnormalities cause the presence of neoantigens, differentiation antigens, or cancer/testis antigens (together termed tumor-associated antigens (TAAs)), which result in the presentation of peptides bound to the major histocompatibility class I (MHC-I), distinguishing cancer cells from the normal cell types 28. At the beginning of cancer-immunity cycle depicted by Chen and Mellman (step 1 of Figure 1) 1, TAAs are released from dying tumor cells and captured by antigen-presenting cells (APCs). The professional APCs mainly include DCs, macrophages and B cells, and among these, DCs play critical roles in starting and regulating the anticancer immunity 29. Subsequently, TAAs are bound to MHC-I/MHC-II of mature DCs (step 2 of Figure 1). When mature DCs migrate into tumor-draining lymph nodes, they present TAAs to T cells, which lead to the priming and activation of effector T cells against TAAs (step 3 of Figure 1). The function of DCs can be regulated by a complex network involving cytokines, chemokines and damage-associated molecular patterns (DAMPs) (Figure 1; also see review in 29).

The phenotypic maturation of DCs is associated with the upregulation of surface markers such as CD80, CD83 and CD86 along with the MHC molecules, whereas the expression of these markers is negative or low in immature or semi-mature DCs 30. When DCs become mature, they secrete medium/high levels of pro-inflammatory or immunostimulatory cytokines (e.g. IL-12, IL-23 and IL-1β) and low level of immunosuppressive cytokines (e.g. IL-10 and TGF-β) 30. In contrast to mature DCs, immature or semi-mature counterparts are devoid of the capacity to prime and activate T cells against tumors, or may even cause T cell anergy and induce tolerance therefore compromising antitumor immunity 29. Recently, the stimulatory and inhibitory factors associated with DC maturation have been used as therapeutic means or targets for developing novel nanomaterial-based strategies.

*Cytotoxic T lymphocytes (CTLs)*: As shown in step 3 of Figure 1, naive CD8 T cells become CTLs when TAAs bound on MHC-I of DCs are interacted with the T-cell receptor (TCR, a disulfide-linked membrane-anchored heterodimeric protein complex composed of CD3 and highly variable alpha and beta chains 31). CTLs are capable of trafficking through tissues (e.g. blood and lymphatic vessels) (step 4 of Figure 1) and infiltrating into tumors (step 5 of Figure 1). It is known that the trafficking and infiltration of CTLs are tightly upregulated by a complex interactions between T cells and endothelial cells, mainly including 31 1) the expression of homing molecules (e.g. PSGL-1 and CD44) on CTLs that can facilitate them to migrate into tumors; 2) a temporary attachment of CTLs onto the endothelium by binding P- and E-selectins via homing molecules; 3) the expression of chemokine receptors (e.g. CXCR3) on CTLs that can bind chemokines (e.g. CXCL9 and CXCL10) released from the TME; 4) the activation of integrins (e.g. LFA-1 and VLA-4) on CTLs that can bind integrin ligands (e.g. ICAM-1 and VCAM-1), which form a firm adhesion between CTLs and the endothelium, leading to extravasation of CTLs into the tumor bed. Subsequently, CTLs release the cytotoxic mediators such as IFN-γ, granzymes or perforin to kill cancer cells in a TCR-dependent manner (steps 6 and 7 of Figure 1).

However, when CTLs enter the TME, they encounter an immunosuppressive milieu, in which inhibitory components derived from tumor cells and stromal cells can affect the phenotype and function of CTLs and finally turn them into “exhausted” state (e.g. decreased proliferation and reduced production of cytotoxic mediators). For example, the activity of CTLs is significantly dampened by immunosuppressive mediators produced by tumor cells such as indoleamine 2,3-dioxygenase 1 (IDO-1), programmed death-ligand 1 (PD-L1), cyclooxygenase type 2 (COX‐2), and signal transducer and activator of transcription 3 (STAT3) 32. In addition, a number of cytokines released from tumor-associated fibroblasts (TAFs), myeloid-derived suppressor cells (MDSCs), macrophage type 2 (M2) cells and regulatory T cells (Tregs) can negatively regulate CTL-mediated cancer killing (step 7 of Figure 1; see below discussion). Therefore, the nanomaterial-based strategies targeting these aforemetioned inhibitory mediators may potentially relieve the exhaustion of CTLs and rescue their cytotoxic function for antitumor immunity.

*T helper (Th) cells*: The cell-mediated antitumor immunity (an immune response that is not involved with antibodies) has been largely attributed to CD8^+^ CTLs, however, emerging evidence indicates that CD4^+^ Th cells also play significant roles in the initiation and maintenance of antitumor effects. When antigens bound onto MHC-II of APCs interact with the TCR, naive CD4^+^ T cells are generally differentiated into Th1, Th2, Th17 and Th9 subtypes 33.

The differentiation of Th1 requires IL-2, IL-12 and IFN-γ, and Th1 cells release IFN-γ, IL-2 and TNF-α for the assistance of CTL differentiation, activation of macrophage type 1 (M1) cells, and mediation of cell-mediated immunity 34. The differentiation of Th2 requires IL-4, IL-6 and IL-10, and Th2 cells secrete IL-4, IL-5 and IL-13 for coordinating humoral immunity (an immune response that is involved with antibodies) (see below discussion). Although Th1 and Th2 subsets are both known to induce antitumor immunity, IFN-γ-secreting Th1 cells have demonstrated better efficacy in this role 33. However, the level of Th2 cytokines within the TME is significantly higher than that of Th1 cytokines, which prevent the production of Th1 cells and activation of CTLs 34. In addition, Th17 cells as an independent CD4^+^ lineage from either Th1 or Th2 have demonstrated a paradox of its function in tumor immunity 35. Although Th17 mediates antitumor immune responses by means of stimulating effector CTLs, they may increase tumor progression through promoting angiogenesis and immunosuppressive events 35. Recently, it has been reported that a subset of CD4^+^ Th cells namely Th9 possess less-exhausted cytolytic function as strong as Th1 cells and demonstrate hyperproliferative feature to persist as long as Th17 cells 36. Th9-mediated anticancer efficacy is highly relied on IL-9 and upregulated expression of Eomes (Eomesodermin; the effector master regulator that controls granzyme expression) and Traf6 (tumor necrosis factor receptor (TNFR)-associated factor 6; one of NF-κB upstream signaling proteins). As a result, tumor-specific Th9 cells eliminated the advanced late-stage melanoma and protected surviving animals against the tumor rechallenge 36, indicating the significant role of Th9 cells in adoptive cancer therapy. As Th subsets are generally supposed as a double-edged sword in tumor immunology, nanomaterial-based therapeutic approaches that balance these Th cells hold the promise for cancer immunotherapy.

*B cells*: The critical contributions of T cells in antitumor immunity have been substantially investigated and well established. In contrast, the immunologic roles of B cells in response to tumors are less well studied. B cells are comprised of functionally distinct subpopulations, and the balance among these has a significant impact on tumoricidal activity 37. When B cells are activated under the stimulation of B cell receptor (BCR) pathway, microRNA pathway, and Toll-like receptor (TLR) pathway 38, they exert antitumor immunity by means of producing antibodies, cytokines and chemokines 39, acting as local APCs 40, and forming tertiary lymphoid structures (TLS, ectopic lymphoid-like structures for long-term antitumor immunity) 41. A subpopulation of B cells termed plasma cells can produce antibodies for antitumor responses, which mainly include antibody-depedent cell-mediated cytotoxicity (ADCC) and complement-dependent cytotoxicity (CDC). In addition, mature follicular B cells (FOB, a subset of B cells) can differentiate into Be-1 and Be-2 cells, which produce cytokines such as IFN-γ, TNF-α, IL-2 and IL-12 for enhancing the antitumor immunity of T and NK cells 39. When stimulated by the CD40-CD40 ligand (CD40L) signaling pathway, B cells become local APCs in tumors, which maintain the survival and proliferation of tumor infiltrating T cells for durable antitumor responses 40. However, B cells are significantly shaped inside the TME, which impair the activity of immunostimulatory B cells but result in differentiation of B cells into an immunosuppressive subtype termed regulatory B cells (Bregs, see below review).

*Natural Killer (NK) cells*: They have long been known as a subclass of cytotoxic lymphocytes that are critical for innate immunity against virus-infected and malignant cells 42. Recently, emerging evidence has displayed that abnormal cells can be distinguished from healthy cells through a group of functional receptors (e.g. inhibitory and activating receptors) on the surface of NK cells 42. The acquisition of corresponding ligands in combination with reduced expression of MHC-I molecules on aberrant cells will exert the cytotoxicity of NK cells against assaults (e.g. viruses and cancers) while ensuring self-tolerance 43. The cytotoxic activity of NK cells is relied on cytokines such as IL-2, IL-12, IL-15 and IFN-α/β 43. The NK cells release IFN-γ to promote the expression levels of MHC-I on cancer cells and MHC-II on APCs, facilitating the connection of innate and adaptive immunities 44. The NK cells are also able to govern the growth and differentiation of DCs and T cells; for example, IFN-γ secreted by NK cells can activate DCs for priming subsequent T-cell responses 43, 44. However, the development of therapeutic means based on NK cells remains a challenge, as the inhibitory factors produced by the TME can significantly cause the dysfunction of NK cells 45. Recent strategies (e.g. checkpoint inhibitors and therapeutic antibodies) have demonstrated the potential to reverse NK cell dysfunction therefore boosting antitumor immunity 46.

*Macrophage type 1 (M1) cells*: Tumor-associated macrophages (TAMs) are a population of immune cells within the TME of solid tumors 47. TAMs are recruited into tumors by chemokines (e.g. CCL2), cytokines (e.g. VEGF, PDGF and M-CSF), and other factors (e.g. fibronectin, fibrinogen, cleavage products of ECM proteins). As two key subtypes of TAMs, the classically and alternatively activated macrophages (termed M1 and M2 respectively) play distinct roles in the processes of immunosurveillance and angiogenesis underlying tumor formation, development, and metastasis 47. When TAMs are under the stimulation of bacterial products (e.g. lipopolysaccharide, LPS) and Th1 cytokines (e.g. IFN-γ and TNF-α), they are driven towards M1. The M1 subtype is normally characterized by immunostimulatory activity and antitumor function 48. For example, M1 cells release Th1 cytokines (e.g. IFN-γ, IL-2 and TNF-α) and chemokines (e.g. CXCL9 and CXCL10) for directly killing tumor cells as well as for indirectly augmenting the cytotoxic activity of T cells 48. In addition, M1 cells are capable of normalizing the tortuous vasculature 49, which can remodel the TME and overcome resistance to cancer therapy. In contrast, M2 cells have a significant impact on tumor progression by promoting genetic instability, supporting tumor growth and metastasis, and orchestrating tumor immunity (see below discussion).

### 2.2. Immunosuppresive cells

*Myeloid-derived suppressor cells (MDSCs)*: It is known that mononuclear cells (monocytes, they are terminally differentiated into macrophages and DCs) and granulocytes (for example, neutrophils as the most abundant representative) originate from hematopoietic stem cells via common myeloid progenitors within the bone marrow (BM) 50. The activity of these myeloid cells is tightly governed by a network of signals from pathogens in the form of TLR ligands, DAMPs and/or pathogen-associated molecular patterns (PAMPs) 50. These signals are often strong but end in a short duration. The response to the signals leads to a rapid mobilization of monocytes and neutrophils from the BM, the significantly enhanced phagocytosis, a generation of pro-inflammatory cytokines, and the upregulation of MHC class II and costimulatory molecules 51. In contrast, the signals generated by chronic conditions (e.g. cancers) are relatively weak but sustain for a long while 52. When the nature of myeloid cells is deformed under cancerous condition, monocytes/neutrophils demonstrate immature phenotype and morphology, ineffective phagocytic activity, and high expression of anti-inflammatory cytokines 53. Consequently, these immature myeloid cells are proliferated and converted to MDSCs. MDSCs always coexist with normal monocytes and neutrophils in cancer patients, but the number of MDSCs is increased during tumor progression and becomes dominant, which suppress the adaptive immunity and facilitate tumor progression and metastasis 53.

MDSCs consist of two main subpopulations namely monocytic (M-) 54 and polymorphonuclear (PMN-) 55 MDSCs. Increasing evidence indicates that M-MDSCs are phenotypically and morphologically similar to monocytes, and PMN-MDSCs are similar to neutrophils 56. M-MDSCs (CD11b^+^Gr1^low^ phenotype) rapidly differentiate into TAMs within tumors, in which these terminally differentiated myeloid cells (most likely macrophage type 2, see below discussion) inhibit immune responses and promote tumor development 56. On the other hand, PMN-MDSCs (often referred as immunosuppressive neutrophils, with a CD11b^+^Gr1^high^ phenotype) are propagated inside tumors in which they become the dominant subpopulation of neutrophils 57. As MDSCs and monocytes/neutrophils share a common number of markers and are identical in morphology, there is still a debate associated with the relationship between these cells. Therefore, future studies are needed to solve the controversy and confusion surrounding the true nature of MDSCs (see review in 58).

The migration of MDSCs to tumors is achieved by chemokines, and among these, CCL2 and CCL5 are considered the main chemokines underlying the MDSC migration 59, 60. The other chemokines such as CCL15, CXCL5, CXCL6, CXCL8 and CXCL12 have also been reported to induce the recruitment of MDSCs into the TME 59. As one of the major cellular components of the TME, MDSCs exert immunosuppressive activities mainly by the upregulation of inhibitory PD-L1 on the surface, release of immunosuppressive cytokines such as transforming growth factor (TGF-β) and IL-10, and production of chemokines (e.g. CCL4 and CCL5) for Tregs into tumors 60. As MDSCs are phenotypically and morphologically similar to monocytes and neutrophils, therapeutic strategies that can specifically target MDSCs may provide better therapeutic benefits (see review in 59, 60).

*Macrophage type 2 (M2) cells*: In contrast to M1 cells that act preferentially in pro-inflammatory responses and antitumor cytotoxic function, M2 counterparts exert anti-inflammatory and tissue remodeling/regenerative roles 61. MDSCs may drive TAMs towards the M2 phenotype by increasing the secretion of IL-10 and alleviating the production of IL-12 48. In addition, when TAMs are infiltrated into tumors, they are preferentially differentiated into M2 cells under the stimulation of cytokines (e.g. IL-4, IL-13, IL-21 and IL-33) and chemokines (e.g. CCL2 and CXCL4) 48. The M2 subclass is functionally characterized by the immunosuppression and the promotion of tissue remodeling (e.g. angiogenesis). For example, M2 macrophages express different chemokines such as CCL17, CCL22 and CCL24, and these chemokine receptors are present on Th2 and Treg cells 48, 61. As such, the release of M2 chemokines can lead to the recruitment of immunosuppressive cells into tumors. The activation of M2 cells exerts inhibitory activity against DCs and T cells by releasing suppressive cytokines (e.g. IL-10 and TGF-β) 62, produces inhibitory metabolites for T cell suppression/anergy/death by triggering IDO-1 mediated pathway 63, and induces immune tolerance by expressing checkpoint molecules (e.g. PD-L1 and CD47) 64. M2 macrophages also facilitate neovascularization by the release of pro-angiogenic mediators such as IL-8, vascular endothelial growth factor (VEGF), basic fibroblast growth factor (bFGF), and epidermal growth factor (EGF) 65. Recently, the inhibition of TAM recruitment and depletion of M2 macrophages have provided therapeutic opportunities to restrain tumor growth and metastasis 66. In addition, due to the controversial (supportive and inhibitory) role of TAMs, strategies reprogramming the phenotype from M2 to M1 to rescue antitumor immunity have presented significant antitumor potential 61.

*Regulatory T cells (Tregs)*: As a subtype of T cells, Tregs play important roles in the maintenance of immunological tolerance in the periphery (e.g. autoimmune diseases) by suppressing the host immunity against self- and nonself-antigens 67. The most physiologically relevant Tregs are characterized by the expression of surface markers CD4/CD25 and transcription factor Forkhead box protein 3 (FoxP3) 68. Accumulating evidence indicates that an elevated number of CD4^+^CD25^+^FoxP3^+^ Tregs are infiltrated into tumors, and their abundant presence is considered a major hurdle to effective immunotherapy 69. It has been reported that Tregs in patients with tumors, as compared to those in healthy populations, are often evident with high expression of chemokine receptors such as CCR4, CCR5 and CXCR4, and the corresponding chemokines derived from the TME can facilitate the infiltration of Tregs into tumors 70. Treg-mediated suppressive mechanisms mainly include: 1) they scarcely produce IL-2 but express the high-affinity IL-2 receptor α chain (CD25) to deprive this cytokine from the neigbour, which may limit the activation and proliferation of effector T cells 71; 2) CTLA-4 expressed on Tregs has higher affinity for CD80 and CD86 (co-stimulatory molecules) on DCs than CD28 expressed on T cells does (the interaction between CD28 and CD80/CD86 provides co-stimulatory signals required for the activation and survival of T cells), thus hindering co-stimulation of T cells 72. In addition, the binding of CTLA-4 with CD80/CD86 may downregulate the expression of these co-stimulatory molecules, further causing the inactivation of T cells 73; 3) Tregs can produce immunosuppressive cytokines (e.g. IL-10 and TGF-β) to skew the function of DCs and T cells, and may even cause direct killing of these immunostimulatory cells by secreting granzymes and perforin 74. Recent progress in tumor immunotherapy targeting Treg-mediated immunosuppressive mechanisms holds great promise for cancer patients 75.

*Regulatory B cells (Bregs)*: As discussed above, B cells are subveted toward Bregs inside the TME, which is accomplished by pathways of TLR, CD40/CD40L, B-cell activating factor (BAFF), BCR, and CD80/CD86 76. Bregs negatively regulate antitumor immunity through different mechanisms: 1) they produce immunosuppressive mediators such as cytokines (e.g. IL-10, TGF-β and IL-35) and IDO-1, which can suppress the proliferation and activation of T and NK cells 40; 2) Bregs inactivate these immunostimulatory cells by expressing immune checkpoints (e.g. PD-L1) 77; 3) when Bregs express the death-inducing molecule Fas ligand (FASL), they will induce the apoptosis of effector T cells 78; 4) Bregs promote tumor progression by secreting TGF-β for epithelial-mesenchymal transition (EMT) 79. In addition, the expression of suppressive markers (e.g. FoxP3 and CTLA-4) on Tregs can be promoted by Bregs by cell-to-cell contact 80. Therefore, strategies used to target or reshape Bregs may provide therapeutic potential for rescuing antitumor immunotherapy.

*Stromal cells*: As important cell types inside the TME, stromal cells (e.g. fibroblasts, vascular endothelial cells and pericytes) usually facilitate the development and maintenance of tumors by supporting tumor cells, remodeling ECM, and promoting angiogenesis 81. Recently, accumulating evidence has indicated that stromal cells also play immunosuppressive roles within the TME 81. As one of the prominent stromal cells inside the TME, tumor-associated fibroblasts (TAFs) are composed of heterogeneous subtypes that are derived from different cellular origins (e.g. local fibroblasts and mesenchymal stem cells) 82. The fibroblasts are usually quiescent in healthy tissues and early-stage cancers, however, they become activated and are turned into TAFs following a serial of physiological and biochemical changes during tumor progression (see review in 83). TAFs are involved in ECM remodeling, tumor immunity, angiogenesis, and cancer cell proliferation and metastasis, which have previously been reviewed 84, 85. In addition, the highly heterogeneous tumor vasculature is also a key component associated with the TME in many solid tumors, which result in abnormal blood flow into under-perfused tumor areas 86. Due to the lack of functional intratumor lymphatic vessels, the elevated interstitial fluid pressure disrupts the transport of therapeutic agents to the TME 86. The tumor blood and lymphatic vascular networks can hinder immunosurveillance mechanisms and suppress antitumor immunity, which have been discussed elsewhere 87. Therefore, novel therapeutic strategies used to remodel these stromal cells also hold great promise for overcoming immunotherapy resistance 87.

Recently, the reprogramming of immunoregulatory cells has been achieved using nanomaterial-based approaches, which can profoundly improve immune therapy against cancers. The methods of engineering nanomaterial-based approaches for targeted modulation of immunoregulatory cells have been extensively reviewed by Shi et al. 88 and Yu et al. 89, demonstrating the significant promise of NPs for enhancing the efficacy of current immunotherapies (see reviews for more details). It is known that the reprogramming of one single cell type is normally not sufficient to achieve antitumor efficacy, whereas the modulation of different cell populations simultaneously may lead to satisfactory therapeutic outcome. Notably, the concepts of immunoregulatory cells still remain debatable due to controversial issues such as the origin and nature of these cells and their distinctive biological roles at different stages of cancer (so called the double-edged sword) 90. Therefore, technologies that precisely discriminate these cells are urgently required to solve these controversies, obtain a deeper insight into the definition of distinctive cell types, and confirm therapeutic targets for nanomaterial-based immunotherapeutics (nanoimmunotherapeutics).

### 2.3. Technologies for characterization and quantification of immunoregulatory cells

As described above, the TME is composed of a heterogeneous population of tumor cells and distinct resident/infiltrating non-tumor cells such as immune cells, fibroblasts, endothelial cells, pericytes 91, and adipocytes 92. Tumorigenesis is profoundly affected by reciprocal interactions between these cells through cell-to-cell contact, secreted factors, and ECM proteins/peptides 6. Recent studies have suggested the impact of resident/tumor-infiltrating host cells on cancer prognosis and clinical outcome of immune-based therapies 9, indicating the importance of immunoregulatory cells in the TME. In addition, a deeper analysis of complexity and diversity of immunoregulatory cells may facilitate a better understanding of how these cells affect the TME, which will enable the prediction of therapeutic responsiveness and reveal new therapeutic targets. The commonly used technologies to identify and quantify immunoregulatory cells in terms of phenotypic and functional analyses are selectively discussed in here.

*Analysis of immunological phenotypes*: As shown in Table 1, immunoregulatory cells represent a heterogeneous population, which differ in their cell surface antigens and intracellular markers in a spatiotemporal manner (e.g. early stage v.s. late stage and tumor-infiltrating v.s. blood circultating). These molecules can be characterized and quantified using antibody-based imaging and cellular phenotypic techniques, such as immunohistochemical (IHC) staining assay 93, immunofluorescent (IF) microscopy 94, and flow cytometry 95. The IHC- and IF-based analyses can be used to study the expression and location of antigens of interest from *in vitro*, *in vivo* and clinical samples. These techniques are also useful to investigate the trafficking, internalization, and recycling of surface antigens/receptors. In addition, the co-localization of cells with cells may also be assessed using these technologies. However, it is worth noting that IHC- and IF-based analyses are often associated with practical pitfalls 96 and subjective interpretation 93, therefore, experienced researchers and qualified pathologists are required to perform experimental procedures and data analyses. Also, it is difficult to track different antigens inside individual cells from the same slice of a sample using IHC- and IF-based analyses. In contrast to these techniques, flow cytometry may provide greater sensitivity and specificity for single cells 95, and therefore has long been considered a preferred analysis method in the field of immunology. Recently, the incorporation of imaging, spectrometric and cytometric technologies including the mass spectrometry IHC (MSIHC) 97, quantitative immunofluorescence (QIF) 98, imaging flow cytometry (IFC) 99 and mass cytometry (flow cytometry coupled with mass spectroscopy) 100, may provide more reliable and reproducible antibody-based technologies for characterization and quantification of immunoregulatory cells. In addition, clinical imaging modalities such as positron emission tomography (PET) and magnetic resonance imaging (MRI) have also been used for the detection of tumor-associated immune cells (e.g. macrophages) in animal models and patients 101.

It is worth noting that although the imaging and cellular phenotypic technologies are widely applied, they can only provide partial information about the “immune fingerprint” due to their limited ability for characterizing a tremendous number of immune subpopulations in tumors. In recent years, bioinformatics, which is defined as a subject that combines biology, computer science, information engineering and mathematics/statistics, has become one of fastest growing technologies in the fields of biology and medicine 102. Bioinformatics has earned its place as a high-throughput computational tool to analyze large collections of biological data (e.g. DNA/RNA sequences, protein samples and cell populations) in a whole genome pattern 103. This technique can be used for discovering novel candidate genes/proteins underlying disease progression as well as for identifying new therapeutic targets 104. Computational genomic tools, which are categorized into two methods namely gene set enrichment analysis (GSEA) and deconvolution, can be used to comprehensively analyze immunophenotype in the TME 105. Both methods are relied on a matrix of expression profiles (e.g. gene expression profiles, DNA methylation profiles or IHC profiles) for individual cell populations, and the detail has been substantially reviewed 105, 106. Among these single-cell analyses, single-cell RNA sequencing (scRNA-seq) has received increasing attention due to its ability to uncover complex and rare cell populations, reveal relationships between genes, and delineate distinct cell lineages during early development 107. By means of isolating individual cells, obtaining the transcripts, and establishing sequencing libraries (the transcripts are mapped to single cells) 108, scRNA-seq also allows researchers to assess highly diverse immune cell populations in healthy and malignant sites/states 109. For example, Szabo et al. utilized scRNA-seq to define the heterogeneity of T cells isolated from the blood, bone marrow, lungs and lymph nodes from healthy donors 110. By analysis of over 50,000 resting and activated T cells throughout these tissues, authors described T cell signatures (e.g. distinct effector states for CD8^+^ T cells and an interferon-response state for CD4^+^ T cells) and generated a healthy baseline dataset 110. Subsequently, the comparison between the scRNA-seq profiles of tumor-associated T cells published by others and the reference map of healthy dataset generated by authors revealed the predominant activities of T cells at different tumor sites, providing insights of how to define the origin, composition and function of immune cells in malignant diseases 110. Therefore, it is expected that the heterogeneity and dynamics of immune cell infiltrates in tumors can also be characterized using scRNA-seq in response to NP-based immunotherapy.

In addition to characterization and quantification between immunoregulatory cells, a variety of computational methods and software tools (see guidelines in 105, 106) may be used to unravel tumor-immune cell interactions for better understanding of tumor immunology, predict neoantigens for therapeutic cancer vaccination, and determine mechanistic principles for combination treatment with synergistic effects 111.

*Analysis of immunological functions*: As shown in Figure 1, immunoregulatory cells produce a variety of stimulatory and suppressive cytokines and chemokines to manipulate the crosstalk between cancer cells and the host immune system. In order to accurately detect and quantitate the immune responses within the TME, a number of techniques such as real-time quantitative polymerase chain reaction (qPCR), enzyme-linked immunosorbent assay (ELISA), enzyme-linked immunospot (ELISPOT) and flow cytometry, can be carried out to evaluate the *in vitro* and *in vivo* expression of cytokines and chemokines. The level of cytokine mRNA transcripts from *in vitro* and *in vivo* models can be measured using qPCR. The* in vitro* and *in vivo* release of cytokines by immune cells may be assessed by either quantifying bulk cytokine production using ELISA 112 or measuring individual cytokine-producing cells using ELISPOT 113. Detection of intracellular cytokines from tumor tissues, lymph nodes and peripheral blood may also be carried out using flow cytometry 114; for example, CD8 and IFN-γ double-positive T cells are considered effector CTLs 115. In addition, immunostimulatory cells will proliferate in response to successful immune-based therapies, whereas immunosuppressive counterparts will decline. The proliferative states of T cells may be evaluated by flow cytometry according to the level of proliferation markers (e.g. Ki67) and the intensity of proliferation tracking fluorescent dyes (e.g. carboxyfluorescein succinimidyl ester (CFSE)) 116.

It is worth noting that these phenotypic and functional analysis technologies have certain limitations (see discussion in 93, 96, 106), therefore, it is critical to understand their ability and availability, in order to assist in the selection of appropriate and accurate ones. In fact, a combination of these techniques is preferred to provide high-accuracy for characterization and quantification of immunoregulatory cells.

## 3. Recent Advances in Nanomaterial-Based Strategies for Cancer Immunotherapy via Modulation of TME

The TME, which contains immunosuppressive cells and soluble signaling molecules, disorganized blood vessels and the dense ECM, is highly resistant to currently available immune-based therapies. Recent advances in the fields of nanotechnology and biomedical engineering provide great potential for the delivery of immunoregulatory agents to modulate the TME systemically (lymph nodes) and locally (tumors) 117, in order to restore the cancer-immunity cycle (Figure 1). Nanomaterial-based delivery strategies designed for immunotherapy, when applied alone or in combination with chemotherapy, gene therapy, phototherapy and radiotherapy, have profoundly revolutionized cancer therapy 118. *In vivo* studies using a variety of immunotherapeutics are summarized in Table 2 according to the material type, nanoformulation strategy, and immunologic modulation. In this section, selected recent examples will be discussed based on the “fuel the engine, release the brake” rules of cancer immunotherapy.

### 3.1. Promoting immunostimulatory effects to “fuel the engine”

**Figure 2:**
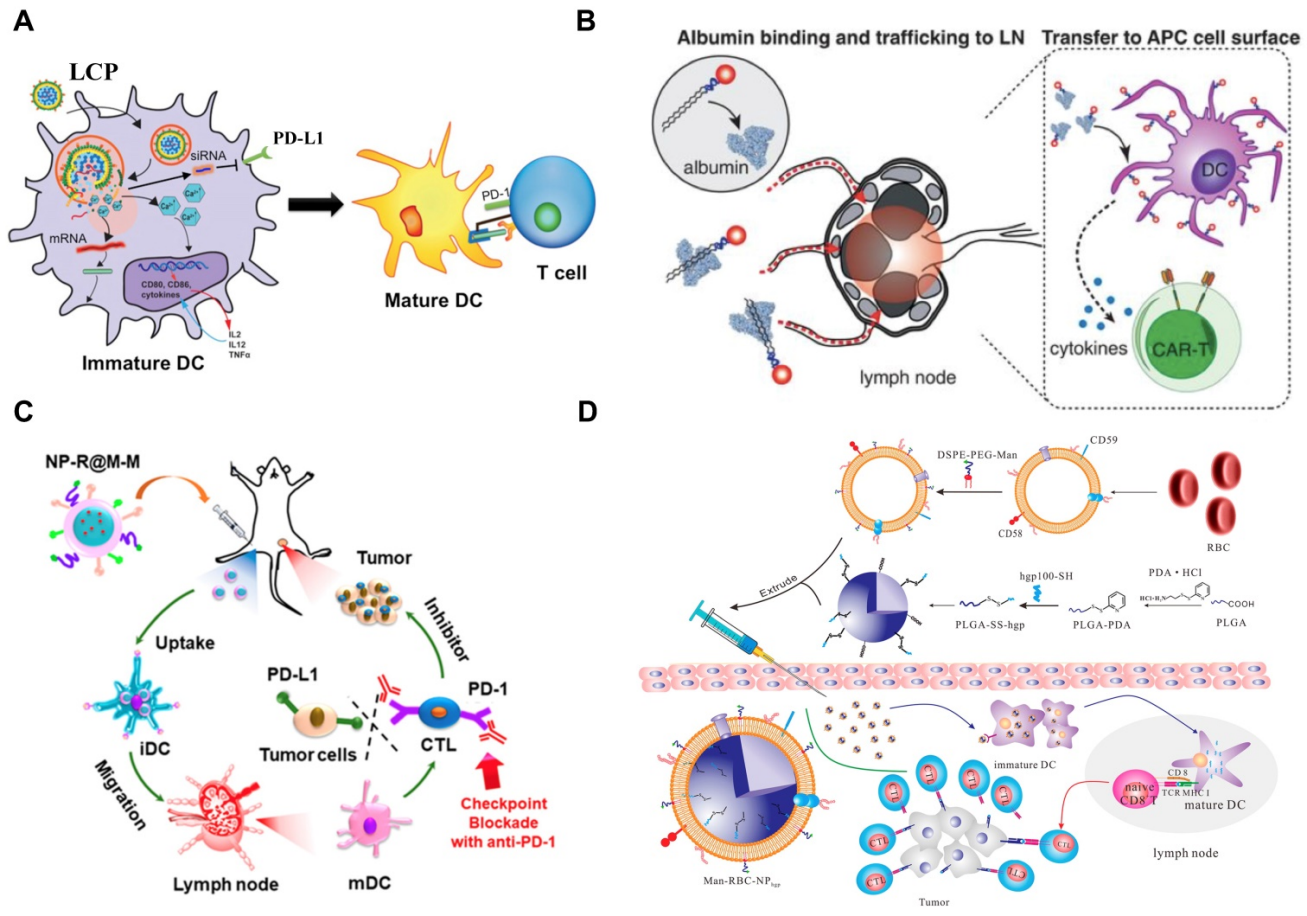
Development of nanovaccines for promoting immunostimulatory effects to “fuel the engine” A) LCP-based delivery of mRNA vaccine for an enhanced immune response against melanoma. Adapted with permission from 160, copyright 2017 Elsevier. B) Albumin-mediated enhanced CAR-T cell activity for solid tumors. Adapted with permission from 150, copyright 2019 American Association for the Advancement of Science C) Cancer cell membrane-coated adjuvant NPs with mannose modification for anticancer vaccination. Adapted with permission from 144, copyright 2018 American Chemical Society. D) Erythrocyte membrane-coated NPs as vaccine for antitumor immunity against melanoma. Adapted with permission from 143, copyright 2015 American Chemical Society.

Methods for the initiation of antitumor immunity including the antigen release, presentation and T cell priming/activation (step 1 to step 3, Figure 1) have been substantially studied (Figure 2). Several nanovaccines are currently investigated in clinical trials for certain solid tumors 88, 89. Recently, biomimetic nanovaccines have been developed for overcoming the barriers involving traditional platforms, by means of improving the stability of antigens, targeted delivery, and long-term release 154-156. The modification of NPs with peptides, proteins and antibodies has also been achieved to produce biomimetic nanovaccines with the enhanced potency, which may allow better reprogramming of immune responses 154-156. The approaches of engineering biomimetic nanovaccines and their application in remodeling the TME for cancer immunotherapy have been extensively reviewed (see more details in 154-157).

Nanomaterials alone or when formulated with antigens in a form as DNA, RNA or peptides can be designed for delivery into APCs in the lymph nodes, which boost T cell priming and activation for antitumor immunity 158, 159. Recently, Wang et al. have developed a mannose-targeted PEGylated lipid-coated calcium phosphate (LCP) NP for co-delivery of mRNA (encoding tyrosinase-related protein 2 (TRP2), a melanoma-associated antigen) and siRNA (targeting PD-L1 mRNA) to DCs in the lymph nodes 160. The LCP-mediated expression of TRP2 in DCs elicited a robust antigen-specific CTL response as well as the production of serum immunoglobulin G against the full-length TRP2 protein in mice with melanoma 160. In addition, the PD-L1 expression in DCs was significantly downregulated by LCP-mediated siRNA, resulting in enhancement of T cell activation and proliferation. Consequently, this LCP nanovaccine remarkably inhibited tumor growth and metastasis 160.

It has been recently reported that stimulator of interferon genes (STING, a signaling molecule) plays a significant role in the regulation of intracellular DNA-mediated IFN-dependent innate immunity 161, demonstrating the potential of STING-mediated cancer immunotherapy. The details of molecular pathways associated with STING, STING agonists/inhibitors, and how to activate STING using nanomaterial-based strategies for cancer immunotherapy have been substantially summarized in 162, 163. Recently, Luo et al. demonstrated a nanovaccine by physical mixture of an antigen and a synthetic polymeric NP (termed PC7A NP) 164. In this study, the delivery of tumor antigens to APCs in the draining lymph nodes was achieved using PC7A NP, resulting in the surface presentation while simultaneously activating STING-dependent type I interferon-stimulated genes 164. As a result, this nanovaccine significantly inhibited the tumor growth in mice with melanoma, colon cancer, and human papilloma virus-E6/E7 cancer 164. In addition, cyclic dinucleotide (CDN) agonists of STING have demonstrated a promising role in the activation of tumor immunogenicity 165. However, the therapeutic efficacy of CDNs, due to the hydrophilicity, negative charge and sensitivity to enzymatic degradation, is limited by *in vivo* delivery barriers. Therefore, Shae and co-workers developed a polymeric NP (polymersome) for enhanced intracellular delivery of 2'3' cyclic guanosine monophosphate-adenosine monophosphate (cGAMP, the endogenous ligand for STING) 166. The resultant formulation (termed STING-NPs) significantly increased the cytosolic activity of cGAMP, promoted the STING signaling in the TME and sentinel lymph nodes, and turned immunosuppressive tumors into immunogenic 166. Consequently, the therapeutic outcomes including the suppression of tumor growth, long-term survival, and induction of immunological memory were successfully achieved by STING-NPs in mice with melanoma 166.

In addition to design of nanovaccines for delivery into APCs in lymph nodes, NPs containing certain therapeutic agents may convert cancer cells into their own vaccine. When tumor cells undergo immunogenic cell death (ICD, also known as immunogenic apoptosis), the DAMPs released by dying tumor cells, which mainly include the exposure of calreticulin (CRT) onto cell surface, secretion of adenosine triphosphate (ATP), and release of high mobility group protein B1 (HMGB1), will activate DCs 167. Consequently, ICD makes the dying cancer cells operate as a vaccine that can trigger a tumor-specific immune response 167. ICD can be induced by certain chemotherapeutic drugs (e.g. anthracyclines, mitoxantrone, oxaliplatin, and bortezomib) 168, physical treatments (e.g. UV irradiation and photodynamic therapy) 169, and oncolytic viruses 170. In addition, the details of ICD-associated signaling pathways, the ICD inducers, and NP-based ICD-mediated cancer immune therapy have been extensively described in 168, 171, 172. Recently, Liu and co-workers have developed an amino ethylanisamide (AEAA, targeting Sigma-1 receptors overexpressed on cancers 173)-targeted PEGylated polymeric NP for co-delivery of mitoxantrone (the ICD inducer) and celastrol (a pentacyclic triterpene extracted from Tripterygium wilfordii) in mice with desmoplastic melanoma. Consequently, the resultant formulation containing two agents at the optimal ratio significantly induced ICD-mediated immunotherapeutic effects, reprogram the fibrotic and immunosuppressive TME, and promote the progression-free survival and sustained immunosurveillance in diseased mice 174.

### 3.2. Overcoming immunosuppressive barriers to “release the brake”

**Figure 3:**
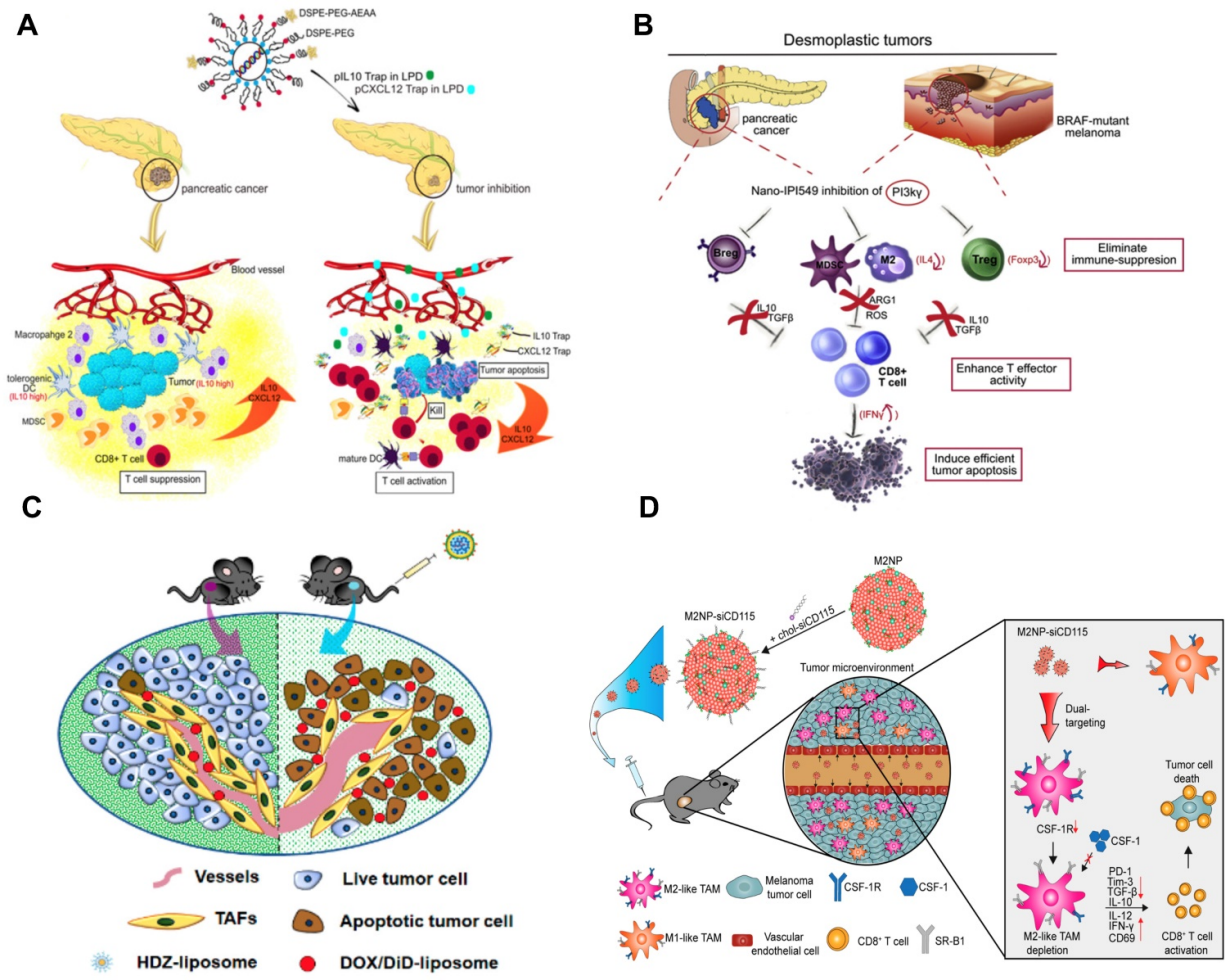
Development of nanoimmunotherapeutics for overcoming immunosuppressive barriers to “releasing the brake”. A) Local blockade of IL-10 and CXCL 12 using LPD for antitumor response for pancreatic cancer. Adapted with permission from 121, copyright 2018 American Chemical Society. B) Inhibiting PI3 kinase-γ using AEAA-targeted PLGA in both myeloid and plasma cells to remodel the suppressive TME in pancreatic cancer. Adapted with permission from 173, copyright 2019 Elsevier. C) Liposome-mediated delivery of vasodilator hydralazine for nanoparticle penetration in advanced desmoplastic melanoma. Adapted with permission from 125, copyright 2019 American Chemical Society. D) Immunotherapeutic strategy for melanoma via dual-targeting NPs delivering siRNA to TAMs. Adapted with permission from 176, copyright 2017 American Chemical Society.

The efficacy of antitumor immunity including the trafficking/infiltration of T cells, recognition of tumor cells by T cells and killing of tumor cells (step 4 to step 7, Figure 1) is significantly dampened by the immunosuppressive TME. Therefore, approaches used to overcome such immune tolerance have been extensively investigated (Figure 3). Recent advances in nanoengineered strategies for delivery of checkpoint inhibitors have been reviewed 175. These NP-based approaches enable the selective delivery of checkpoint inhibitors into tumors, which can reduce immune-related toxic issues. Consequently, they significantly reprogram immunosuppressive cells and improve the activity and persistence of effectors T cells. In addition, a number of NP-based delivery approaches have been recently developed for delivery of therapeutic components (e.g. chemotherapeutics, antibody and siRNA) to target immunosuppressive soluble mediators such as TGF‐β, IDO, COX‐2 and epidermal growth factor receptor (EGFR), which can significantly remodel the suppressive TME and restore the antitumor effects with reduced systemic toxicity (see review in 175).

TAMs have recently become a promising therapeutic target; however, it is still challenging to deliver therapeutic agents to them. Recently, a liposomal NP has been developed with the modification of α-peptide (a scavenger receptor B type 1 (SR-B1) targeting peptide) and M2pep (an M2 macrophage binding peptide) 176. Following intravenous (i.v.) injection this dual-targeted NP demonstrated higher binding affinity to M2-like TAMs than to tissue-resident macrophages in healthy tissues. As a result, the inhibition of survival signals in M2-like macrophages as well as the depletion of this cell type from melanoma were achieved using this dual-targeted NP containing siRNA against colony stimulating factor-1 receptor (CSF-1R), which was observed along with the increase of immunogenic cytokines (IL-12 and IFN-γ) and reduction of immunosuppressive cytokines (IL-10 and TGF-β) 176. In addition, Rodell et al. developed a β-cyclodextrin NP (CDNP) for delivery of R848 (an agonist of TLR7 and TLR8) in a range of tumor models in mice 177. As a result, CDNP-R848 significantly altered the TAMs toward the M1 phenotype, which slowed down tumor growth and protected mice against tumor rechallenge 177. More importantly, improved antitumor immune responses were achieved by CDNP-R848 when applied in combination with anti-PD-1 therapy, confirming the potential of NP-based strategies to effectively remodel TAMs for cancer immunotherapy 177.

Stromal cells as one of the key cellular components in the TME usually facilitate the development and maintenance of tumors by supporting tumor cells, remodeling ECM, and promoting angiogenesis 81. It has been reported that the development of liver metastasis is often associated with activated hepatic stellate cell (aHSC)-mediated liver fibrosis, and the relaxin (RLN, an anti-fibrotic peptide) can deactivate aHSCs and therefore resolve liver fibrosis 122. Therefore, an AEAA-targeted PEGylated LCP NP containing the RLN plasmid was developed by Hu and co-workers to target cancer cells and aHSCs within the metastatic lesion and use them as an *in situ* factory for the production of RLN protein. Consequently, the stromal microenvironment in liver metastases was effectively reversed by LCP-mediated expression of RLN protein, which significantly inhibited metastatic progression and prolonged the survival of animals, accompanied with the upregulation of immunogenic cells/cytokines and downregulation of immunosuppressive counterparts 122.

Although NPs may take advantage of the enhanced permeability and retention (EPR) effect for tumor accumulation 178, the elevated interstitial fluid pressure, high density of ECM and disorganized blood vessels (particularly in desmoplastic tumors) cause significant hurdles for particle penetration. To address these issues, Chen and colleagues developed a hydralazine (HDZ, a routine medication used to treat high blood pressure and heart failure)-containing liposomal NP to reshape tumor blood vasculature in advanced desmoplastic melanoma 125. The i.v. injection of HDZ-liposome favorably modulated the vascular dilation, tumor hypoxia, and tumor permeability, which were accompanied with the TME modulation (Figure 3). As a result, the HDZ-liposome significantly improved the therapeutic efficacy of liposomal doxorubicin as the second-wave treatment in mice with tumor size over 400 mm^3^ 125. In addition, it has been reported that high concentration of perivascular nitric oxide (NO) can facilitate tumor vascular normalization and further the chemotherapy efficacy 130. Despite the promising anticancer effect, the clinical application of NO is limited by the short half-life, low bioavailability, and poor tumor targeting behavior 130. Recently, Sung et al. have developed a poly(lactic-co-glycolic acid) (PLGA)-based delivery system (NanoNO) containing dinitrosyl iron complex (DNIC, the NO donor) 130. In murine hepatocellular carcinoma model, NanoNO was able to provide sustained NO release into tumors, which resulted in effective normalization of tumor vasculature and improve the delivery of follow-up chemotherapy for the suppression of primary tumors and metastases 130. Immunological analyses revealed that NanoNO at a lower dose could reprogram the immunosuppressive TME therefore improving the anticancer efficacy 130.

### 3.3. The combination therapy

**Figure 4:**
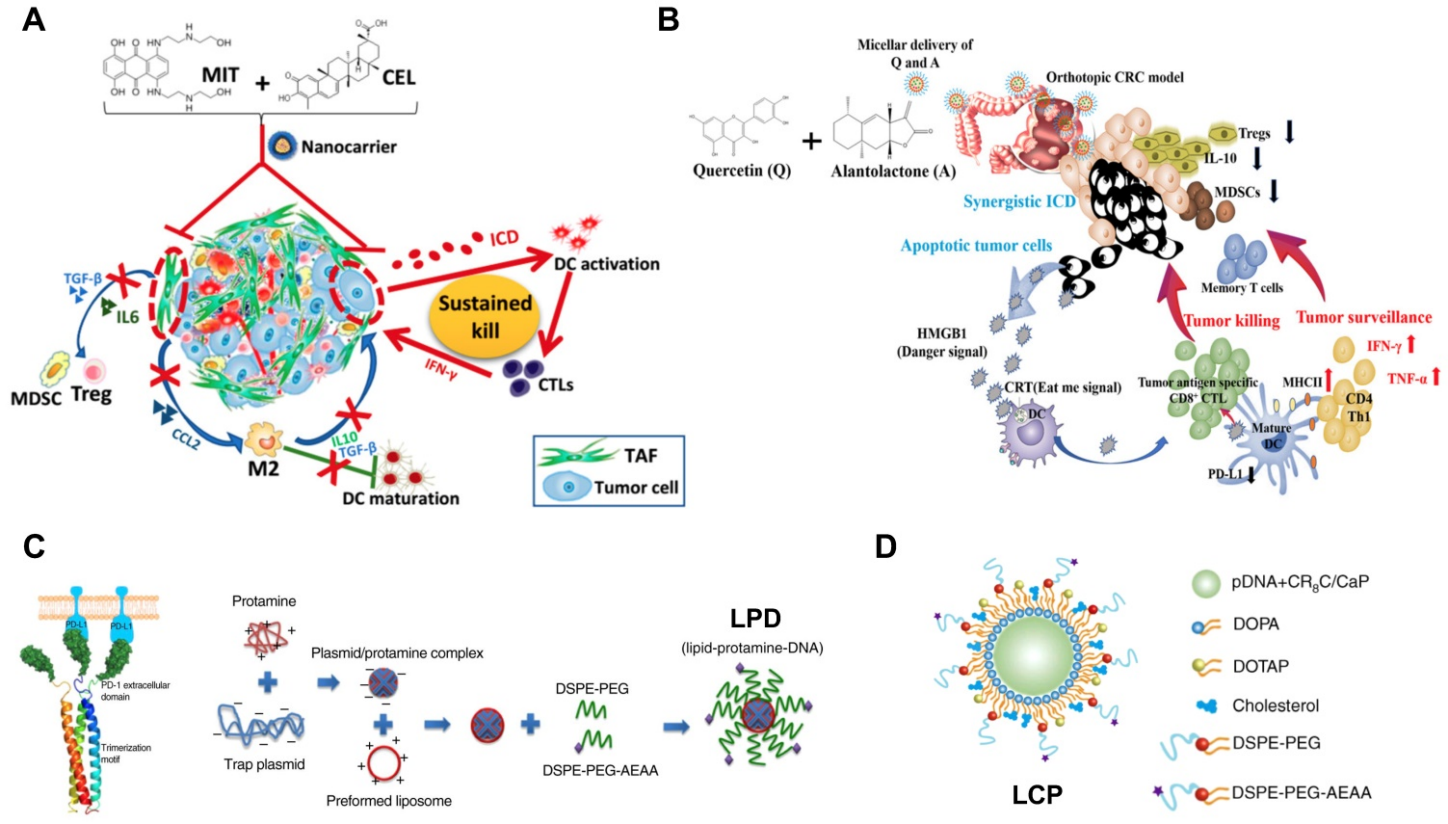
Development of nanoimmunotherapeutics for combination therapy. A) NP-mediated co-delivery of mitoxantrone (MIT) and celastrol (CEL) to induce chemo-immunotherapy for cancer inhibition and tumor dormancy in desmoplastic melanoma. Adapted with permission from 174, copyright 2018 American Chemical Society. B) NP-based co-delivery of Quercetin (Q) and Alantolactone (A) for antitumor responses through synergistic ICD. Adapted with permission from 131, copyright 2019 American Chemical Society. C) Synergistic and low adverse effect cancer immunotherapy by LPD-mediated immunogenic chemotherapy and locally expressed PD-L1 trap in combination with oxaliplatin for colorectal cancer. Adapted with permission from 119, copyright 2018 Nature Publishing Group. D) LCP-mediated relaxin gene delivery for synergistic effect with checkpoint inhibition in liver metastasis. Adapted with permission from 122, copyright 2019 Nature Publishing Group.

Schemes that simultaneously target stimulatory and inhibitory mechanisms potentially provide synergistic antitumor immunotherapeutic effectiveness (Figure 4). It has been reported that the blockage of immune checkpoint molecules using systemically administrated mAbs may reverse the immune tolerance, but autoimmune-like side effects are unavoidable for healthy tissues or organs 179. Alternatively, local delivery of immune checkpoint inhibitors in the TME may alleviate the immune-related adverse effects (irAEs). Therefore, Song and colleagues developed an AEAA-targeted lipid-protamine-DNA (LPD) NP for delivery of plasmid encoded with PD-L1 trap (a small antibody-like fusion protein targeting PD-L1) in mice with colorectal cancer. Consequently, the expression of PD-L1 trap by LPD in tumors led to a synergistic chemo-immunotherapeutic outcome in combination with oxaliplatin (OxP)-mediated ICD effects, resulting in longer animal survival time and lower level of irAEs, in comparison with free PD-L1 mAb and OxP 119.

It is known that IDO‐1 is one of tryptophan catabolic enzymes that can facilitate the conversion of tryptophan (Trp) to kynurenine (Kyn) 180. The downregulation of Trp can suppress the proliferation and activity of CTLs and NKs, and the upregulation of Kyn can activate Tregs and MDSCs 181. Therefore, approaches against IDO-1 hold great promises for tumor immunotherapy. Indeed, the combination immunotherapy has been achieved using the co-delivery of IDO-1 inhibitor and immune checkpoint inhibitor 182. In addition, it has been reported that IFN-γ released by ICD-mediated CTLs can positively regulate tumor immunogenicity, but may also cause the production of IDO-1, which dampen the immunotherapeutic efficacy 183. To address such paradox, Feng et al. developed an amphiphilic polymeric NP for co-delivery of OxP prodrug and NLG919 (an IDO-1 inhibitor) to induce OxP-mediated ICD effects and reverse IDO-1 mediated immunosuppression, respectively 133. Consequently, the resultant nanoformulation (BCPN) could achieve significantly better tumor inhibition at primary and metastatic sites than the combination of free OxP and NLG919 133.

In addition, a light-sensitive *in situ* gelation system was reported by Meng et al. for the combination of photodynamic therapy and immunotherapy 184. In this study, the photosensitizer (Chlorin e6, Ce6) modified-catalase (CAT, an enzyme triggers the rapid decomposition of H_2_O_2_) was conjugated with poly(ethylene glycol) double acrylate (PEGDA) to form Ce6-CAT-PEGDA. Subsequently, the Ce6-CAT-PEGDA was mixed with imiquimod (R837)-loaded PLGA NPs (RPNPs, the immune adjuvant), forming a polymeric matrix (Ce6-CAT-PEGDA-RPNPs) 184. When locally applied to tumors and exposed under 660 nm red light, Ce6-CAT-PEGDA-RPNPs significantly reversed the immunosuppressive TME by the production of O_2_ that can relieve the tumor hypoxia 184. Consequently, the photodynamic therapy-mediated ICD together with immune adjuvant could mediate a significantly stronger “abscopal effect” for tumor inhibition at primary and distant sites 184.

## 4. Conclusions and Future Perspectives

In recent years, an improved understanding of cancer biology 185 and the discovery of cellular and molecular mechanisms for innate and adaptive immunologic responses 186 have significantly revolutionized the fields of cancer immunology and immunotherapy. These have remarkably encouraged researchers to investigate the possibility of restoring the cancer-immunity cycle using nanomaterial-based immunotherapeutics (nanoimmunotherapeutics) 187-189. Several studies of NP-based cancer immunotherapy are currently undertaken in clinical trials (see the summaries in 88, 89). Despite the potential of nanoimmunotherapeutics for solid tumors 89, none of them have reached the clinic for patients. One major reason for the lack of clinical translation is the presence of the immunosuppressive TME. As shown in Table 2, substantial studies have been undertaken for investigating the capacity and availability of NP-based delivery of immunoregulatory agents to systemically and locally modulate the suppressive milieu within the TME. These works provide proof of concept for NP-based TME-modulating methods and illustrate the potential of nanoimmunotherapeutics to advance the “fuel the engine, release the brake” rules (see reviews in 175, 190-192).

In addition, one of the major remaining challenges associated with clinical translation of nanoimmunotherapeutics (nanomedicine as well) is still the lack of efficient, safe and widely applied delivery strategies to facilitate the transport of therapeutic agents to tumor sites following systemic administration 193. Although NPs may accumulate into tumors following the EPR effect, the delivery efficacy is extremely low 194. In addition, a large number of intratumoral NPs may be either isolated by the ECM or taken up by non-specific cells. The high density of ECM and tortuous blood vessels (particularly in desmoplastic tumors) may be overcome by NPs containing a variety of TME modulators 122, 125, 130, 195, 196, which relieve the harsh niches associated with the failure of drug delivery and enhance the follow-up treatment of targeted nanoimmunotherapeutics that act specifically in cells of interest.

In addition, it should be borne in mind that complicated modifications of nanomaterials, which is hoped to achieve multifunctional delivery formulations with stabilizing groups, targeting ligands and bioresponsive linkers, may complicate the large-scale and reproducible production. In addition, such extensive modifications may also cause unexpected toxicity. Therefore, further investigation must be performed to keep balance between the therapeutic benefit, the complexity of formulation preparation/scale-up and the risk of toxicity before nanoimmunotherapeutics can be satisfactorily applied for cancer patients.

## Figures and Tables

**Table 1:** The commonly used phenotypic markers for immune cells within the TME in terms of stimulatory/suppressive roles.

Cell Subtypes	Markers	Ref.
Immunostimulatory		
DCs	CD11b^+^ MHCII^+^	14
Cytotoxic T cells	CD3^+^ CD8^+^	15
Helper T cells	CD3^+^ CD4^+^	16
Memory T cells	CD44^+^ CD62L^+^ CD3^+^	17
Follicular B cells	IgD^+^CD21^+^CD22^+^ CD23^+^	18
Plasma cells	CD138^+^CD38^+^	19
Memory B cells	CD20^+^CD27^+^CD40^+^CD80^+^	20
NK cells	CD16^+^ CD56^+^ CD57^+^ NK1.1^+^/NK1.2^+^	21 22
M1 cells	F4/80^+^ CD86^+^ CD80^+^	23
Immunosuppressive		
MDSCs	CD11b^+^ Gr-1^+^	24
M2 cells	F4/80^+^ CD206^+^ CD163^+^	23
Tregs	CD3^+^ CD4^+^ CD25^+^ Foxp3^+^ TIM-3^+^	25 26
Bregs	CD19^+^ IL-10^+^	27

**Table 2:** A brief summary of *in vivo* studies on delivery of immunoregulatory agents using nanoparticles and natural carriers, including material types, nanoformulation strategy, and immunologic modulation. (↑ = upregulation, ↓ = downregulation)

Material type	Nanoformulation strategy	Immunologic modulation	Ref.
Lipids & Liposomes	LPD with PD-L1 trap for colorectal cancer	DC, CD8^+^, CD4^+^ and Memory T ↑ Th17 ↓	119
	LPD with pLPS trap for colorectal cancer	DC, CD8^+^ and CD4^+^ T, M1/M2 ↑ Treg, MDSC ↓	120
	LPD with IL-10 and CXCL12 traps for pancreatic cancer	DC, CD8^+^ T, NK ↑ M2, MDSC ↓	121
	LCP with pRLN for liver cancer	DC, CD8^+^ and CD4^+^ T, M1/M2 ↑ Treg, TAF, MDSC ↓	122
	LCP with CXCL12 trap for liver metastasis	CD8^+^ T ↑ Treg, MDSC, TAF ↓	123
	LCP with BRAF peptide for melanoma	DC, CD8^+^ T, M1/M2 ↑ Treg ↓	124
	Liposome with HDZ to increase NP tumor penetration in desmoplastic melanoma	DC, CD8^+^ and CD4^+^ T, NK, M1/M2 ↑ MDSC, TAF ↓	125
	Lipid NP with OxP and DHA for colorectal cancer	DC, CD8^+^ and Memory T, M1↑	126

Polymers	PMP/OVA/siRNA nanovaccine for melanom	DC, CD8^+^ and CD4^+^ T ↑ Treg, MDSC ↓	127
	AC-NP for melanoma	DC, CD8^+^ T, CD8^+^ T/Treg, CD4^+^ T/Treg ↑	128
	PLGA-R847@Cat NP enhanced radiotherapy for colon cancer	DC, CD8^+^ and CD4^+^ T ↑ Treg, M2 ↓	129
	NanoNO to normalize tumor vasculature for liver cancer	CD8^+^ and CD4^+^ T, M1 ↑ TAF, M2 ↓	130
	TPGS-based nanoemulsion with quercetin and alantolactone for colorectal cancer	DC, NK, CD8^+^ and CD4^+^ T ↑ Treg, MDSC ↓	131
	DINP with aPD1 and aOX40 for melanoma	CD8^+^ and memory T ↑	132
	BCPN with oxaliplatin prodrug and NLG919 for colorectal and breast cancers	DC, CD8^+^ T ↑ Treg ↓	133
	H1-NB NP with OVA for melanoma	DC, CD8^+^ T ↑	134
	Cellax NP with DTX for metastatic pancreatic cancer	TAF ↓	135

Inorganic materials	CaCO_3_ NP gel with aPD-1 and zebularine for melanoma	DC, CD8^+^ and CD4^+^ T ↑ MDSC ↓	136
	CaCO_3_ NP gel with aCD47 for melanoma	CD8^+^ T, M1 ↑ Treg, M2, MDSC ↓	137
	H-MnO_2_ NP for TME modulation for triple negative breast cancer	CD8^+^ T, M1 ↑ Treg, M2 ↓	138
	Fe_3_O_4_-ZnO nanovaccines for colorectal cancer	DC, CD8^+^ and CD4^+^ T ↑	139
	Hollow mesoporous silica nanosphere as cancer immunoadjuvant for lung cancer	CD8^+^ and CD4^+^ T ↑	140
	AuNP-DNA photothermal immunotherapy for tumor	DC, HSP70 ↑	141
	MoS2-PEG-CpG for photothermal cancer immunotherapy	DC ↑	142

Cell membrane coated system	Erythrocyte membrane coated NP as cancer vaccine for melanoma	DC, CD8^+^ T ↑	143
	Cancer cell membrane-coated NP as cancer vaccine for melanoma	DC, CD8^+^ T ↑	144
	Cancer cell membrane-coated NP for anticancer vaccine for melanoma	DC, CD8^+^ T ↑	145
	NP coated bacterial as oral DNA vaccines for melanoma	CD8^+^ and CD4^+^ T ↑	146

Natural carrier mimics	Lipoprotein NP for antigen delivery for colorectal cancer and melanoma	CD8^+^ , CD4^+^ and memory T ↑	147
	Lipoprotein NP with DOX for colorectal cancer	DC, CD8^+^ T ↑	148
	T cells conjugated with IL-15 and IL-21 loaded NP for melanoma	CD8^+^, CD4^+^ and memory T ↑	149
	T cells with amphiphilic ligands for melanoma and glioma	CD8^+^ and CD4^+^ T ↑	150
	T cells conjugated with NSC-87877 loaded NP for prostate cancer	CD8^+^ T ↑	151
	Platelets loaded aPD-L1 for melanoma and triple negative breast cancer	CD8^+^ and CD4^+^ T ↑ Treg ↓	152
	Photothermal therapy for tumor infiltration and antitumor activity of CAR T Cells in melanoma	CD8^+^ and CD4^+^ T ↑	153

## Abbreviations

ADCC: antibody-dependent cell-mediated cytotoxicity
AEAA: amino ethylanisamide
aHSC: Activated hepatic stellate cell
APC: antigen-presenting cell
ATP: adenosine triphosphate
BAFF: B-cell activating factor
BCR: B-cell receptor
bFGF: basic fibroblast growth factor
Breg: regulatory B cell
CAR: chimetic antigen receptors
CCL2: C-C motif chemokine ligand 2
CDC: complement-dependent cytotoxicity
CDN: cyclic dinucleotide
CD40L: CD40 ligand
PD-1: programmed cell death protein 1
Ce6: chlorin e6
CFSE: carboxyfluorescein succinimidyl ester
cGAMP: 2'3' cyclic guanosine monophosphate-adenosine monophosphate
COX-2: cyclooxygenase type 2
CRT: calreticulin
CSF-1R: colony stimulating factor-1 receptor
CTL: cytotoxic T lymphocyte
CTLA-4: cytotoxic T lymphocyte-associated protein 4
CXCL9: C-X-C motif chemokine ligand 9
CXCL10: C-X-C motif chemokine ligand 10
DAMP: damage-associated molecular pattern
DC: dendritic cell
DNIC: dinitrosyl iron complex
ECM: extracellular matrix
EGF: epidermal growth factor
ELISA: enzyme-linked immunosorbent assay
ELISPOT: enzyme-linked immune absorbent spot
EMT: epithelial-mesenchymal transition
EPR: enhanced permeability and retention
FASL: Fas ligand
FOB: follicular B
Foxp3: forkhead box protein 3
GSEA: gene set enrichment analysis
HDZ: hydralazine
HMGB1: high mobility group protein B1
ICAM-1: Intercellular adhesion molecule 1
ICD: immunogenic cell death
IDO-1: indoleamine 2,3-dioxygenase 1
IF: immunofluorescent
IFC: imaging flow cytometry
IFN-γ: interferon gamma
IHC: immunohistochemical
IL: interleukin
irAE: immune-related adverse effect
Kyn: kynurenine
LCP: lipid-coated calcium phosphate
LFA-1: lymphocyte function associated antigen 1
LPD: lipid-protamine-DNA
LPS: lipopolysaccharide
M-CSF: macrophage colony stimulating factor
MDSC: macrophages and myeloid-derived suppressor cell
MHC-I: major histocompatibility class I
MHC-II: major histocompatibility class II
M-MDSC: monocytic-myeloid derived suppressor cell
MRI: magnetic resonance imaging
MSIHC: mass spectrometry IHC
M1: macrophage type 1
M2: macrophage type 2
NK: natural killer
NO: nitric oxide
NP: nanoparticle
OxP: oxaliplatin
PDGF: platelet-derived growth factor
PD-L1: programmed death-ligand 1
PET: positron emission tomography
PLGA: poly (lactic-co-glycolic acid)
PMN-MDSC: polymorphonuclear-myeloid derived suppressor cell
PSGL-1: p-selectin glycoprotein ligand 1
QIF: quantitative immunofluorescence
qPCR: quantitative polymerase chain reaction
RLN: relaxin
scRNA-sq: single-cell RNA sequencing
SR-B1: scavenger receptor B type 1
STAT3: signal transducer and activator of transcription 3
STING: stimulator of interferon gene
TAA: tumor associated antigen
TAF: tumor-associated fibroblast
TAM: tumor associated macrophages
TCR: T-cell receptor
Th: T helper
TGF-β: transforming growth factor beta
TME: tumor microenvironment
TNF-α: tumor necrosis factor alpha
TLR: Toll-like receptor
TLS: tertiary lymphoid structure
Treg: regulatory T
TRP2: tyrosinase-related protein 2
Trp: tryptophan
VCAM-1: vascular cell adhesion molecule 1
VEGF: vascular endothelial growth factor
VLA-4: very late antigen 4
